# Description and evaluation of a bench porcine model for teaching surgical residents vascular anastomosis skills

**DOI:** 10.1186/1756-0500-3-189

**Published:** 2010-07-13

**Authors:** Philipe N Khalil, Axel Kleespies, Markus Rentsch, Wolfgang E Thasler, Karl-Walter Jauch, Christiane J Bruns

**Affiliations:** 1 Department of Surgery - Campus Innenstadt, Ludwig-Maximilians University, Nußbaumstraße 20, 80336 Munich, Germany; 2 Department of Surgery - Campus Großhadern, Ludwig-Maximilians University, Marchioninistraße 15, 81377 Munich, Germany

## Abstract

**Background:**

Numerous models, of variable quality, exist to impart the complex skills required to perform vascular anastomosis. These models differ with regard to the kinds of materials used, as well as their sizes, the time needed for their preparation, their availability, and the associated costs. The present study describes a bench model that uses formalin-fixed porcine aorta, and its evaluation by young surgical residents during a recent skills course.

**Findings:**

The aortic segments used were a by-product of slaughtering. They were fixed and stored after harvesting for eventual use. Ten young surgical residents participated, and each performed one end-to-side vascular anastomosis. The evaluation was a questionnaire maintaining anonymity of the participant containing questions addressing particular aspects of the model and the experiences of the trainee, along with their ratings concerning the need for a training course to learn vascular anastomosis techniques. The scoring on the survey was done using a global 6-point rating scale (Likert Scale). In addition, we ranked the present model by reviewing the current literature for models that address vascular anastomosis skills.

The trainees who participated were within their first two years of training (1.25 ± 0.46). A strong agreement in terms of the necessity of training for vascular anastomosis techniques was evident among the participating trainees (5.90 ± 0.32), who had only few prior manual experiences (total number 1.50 ± 0.53). The query revealed a strong agreement that porcine aorta is a suitable model that fits the needs for training vascular anastomosis skills (5.70 ± 0.48). Only a few bench models designed to teach surgical residents vascular anastomosis techniques were available in the literature.

**Conclusions:**

The preparatory and financial resources needed to perform anastomosis skills training using porcine aorta are few. The presented bench model appears to be appropriate for learning vascular anastomosis skills, as rated by the surgical trainees themselves.

## Introduction

The various suture techniques for performing anastomoses of different types are among the most important skills general surgical trainees need to acquire during their first years of residency. Severe complications can occur if an anastomosis is poorly performed, including insufficiency or stenosis. In the case of an intestinal anastomosis, this will most likely require a re-operation for peritonitis due to drainage of intraluminal contents into the peritoneal cavity or for symptoms of obstruction. Similarly, poorly-performed vascular anastomoses will require additional suturing if bleeding occurs after the release of the clamps; otherwise, in a postoperative vascular stenosis of the anastomosis with a higher risk for early vascular occlusion on the anastomotic side can result.

It is common sense that technical skills affect outcomes, and it is well recognised that there is variability in surgical outcomes among surgeons [1]. Therefore, it is important that surgeons become familiar with single-suture techniques during early residency before applying them in a live patient [2]. The required expertise includes both the theoretical background necessary decision-making in different circumstances as well as the manual techniques, which cannot be generated only during operative assistance. Technical ability is one of the most important components of surgical competency besides decision-making skills [3]. The latter can be learned and assessed by simulation training and via oral and written examinations, whereas the technical surgical skill can go untended [3].

There are various models described in literature that can be used to impart the complex motor skills needed for vascular suturing as well as to assess the particular results [3-17]. These models use different materials, such as artificial substances like silicone, Gore-Tex, latex tubes or PTFE (polytetrafluorethylene) grafts; animal parts available as food, such as chicken wings and legs, and turkey neck or breads; and animal limbs and organs, such as pig hearts coronaries or omentum vessels. Moreover, vegetable leaves, human placenta vessels or umbilical cords and cadaver arms have been proposed for use in training in vascular anastomosis. However, most of these models are for training in microvascular anastomoses only due to their sizing in terms of the vascular diameter (as recently reviewed by Ilie et al.) while only few models can be used to train for larger vascular anastomoses appropriate for general or vascular surgical residents [4]. We have given, for several years, a one-day skills course to teach vascular anastomosis using formalin-fixed porcine aorta in order to provide young surgical residents with the basic set of skills needed.

The present paper aimed to describe, as well as assess and discuss, this bench model, which has some obvious advantages compared to other models described in the literature. Therefore, a recent evaluation obtained from surgical residents being trained in vascular anastomosis techniques at the University of Munich Department of Surgery is provided, along with a literature search for models teaching vascular anastomosis skills.

## Materials and methods

### Course Module Description

The present study was conducted at a surgical skills training session recently held at the Department of Surgery, Campus Großhadern Ludwig-Maximilians University, Munich. The course itself is one of many frequently held surgical hands-on training courses that our department has offered for several years and that are open to all surgical trainees. The evaluation was conducted prospectively using an anonymous questionnaire. The target group were residents who were within their first years of training. The skills session was opened with a lecture that addressed the theoretical essentials of vascular suturing. The course segment of training vascular anastomosis was scheduled for one hour. The anastomoses were performed using an end-to-side technique. Participants were paired to form teams, and the pairs assisted each other during the vascular anastomosis. Two experienced surgical instructors taught the course and provided guidance for the trainees. Feedback was given to every resident during surgery as well as after the anastomosis was performed. Thus, the surgical performance, in terms of handling the instruments and needle, was assessed during the suturing of the anastomosis. Instructions and individualized verbal feedback by the two instructors was possible since the skills training included only ten participants working in groups of two not starting suturing the vascular anastomosis at the same time. The resulting anastomosis was assessed by the distance between the single stitches, and the entry and exit on either side of the tissue were discussed in detail.

### Model Description, Preparation, and Course Equipment

Formalin-fixed porcine aortic segments were used to impart vascular anastomosis skills. They were supplied by Covidien (Covidien, Germany GmbH, 93333 Neustadt a.d. Donau, Germany) who unrestrictedly supported this educational module. However, in general, fresh porcine aortas can be obtained from any urban slaughterhouse and stored in formalin until needed. No further tissue preparation is required. The trainees were supplied with gloves and the necessary surgical instruments (forceps, needle holder and scissors) and monofilament suture material (5-0, double-armed).

### Evaluation, Literature Search and Statistics

A questionnaire maintaining anonymity of the participant containing several questions addressing particular aspects of the course and its content was distributed at the beginning of the course. Questions were divided into those that were answered prior to and after the session. The scoring on the survey was done using a global 6-point rating scale (Likert Scale) ranging from very bad/strong rejection (1) to very good/strong agreement (6), as appropriate. The literature was searched for models for teaching vascular anastomosis techniques using the key words *vascular anastomosis, skills training, models, vascular surgery, teaching and education *in a PubMed query. For data analysis, PASW Statistics 17.0 (Predictive Analytics Software, SPSS Inc., Chicago, IL) was used. All data presented are given in terms of mean value ± standard deviation (SD) or range, as appropriate. N refers to the number of participants who answered a particular question. The photographs presented were taken during the actual course evaluation using a digital camera.

## Results

Ten residents within the first two years of residency (1.25 ± 0.46 years) were trained in suturing vascular anastomoses and also completed the evaluation survey. All but two participants are currently in training at universities, while one was from a regional hospital and one was from a specialised hospital.

### Porcine Aortic Model

The formalin porcine aortic segments were found to be of good quality. No extra preparation was necessary.

### Teaching Course Survey

The analysis of the questionnaire revealed that the residents strongly agreed that the initial didactic course on anastomoses techniques was necessary when asked prior to the teaching (5.90 ± 0.32). This strong agreement is understandable considering that the class reported few manual experiences with regard to vascular anastomosis (total number 2.10 ± 1.29). The class reported to have performed from 1-2 (1.50 ± 0.53) vascular anastomoses in humans prior to this teaching session. After the teaching was finished we again asked for the necessity of such a training module, and the class answered this with the same strong agreement compared to the pre-course query (5.90 ± 0.32). Moreover, the trainees were asked for the quality of the model and whether porcine aortal tissue is suitable to train vascular anastomosis. Here, the residents strongly agreed that porcine aorta fits their needs and is suitable for training (5.70 ± 0.48). The course module presented here was found to be demanding by the trainees (4.90 ± 0.99). The introductory tutorial on the suture technique was rated comprehensible and important to the trainees (5.30 ± 0.48 and 5.00 ± 0.66, respectively). Finally, the participants rated the course module and the time management (3-point rating scale) with high acceptance that it was what they needed (5.60 ± 0.70 and 1.80 ± 0.42). A summary of the pre and post-course survey is given in *Table *1 and 2.

**Table 1:** Essential results of the pre-course survey

	N	Range	Mean	SD
How would you rate your experience regarding vascular anastomosis?	10	1.00 - 5.00	2.10	1.29
How would you rate your theoretic knowledge regarding suture material and suture techniques?	10	1.00 - 5.00	3.40	1.17
How many vascular anastomosis have you performed?*	10	1.00 - 2.00	1.50	0.53
I consider a course to train vascular anastomosis techniques as important	10	5.00 - 6.00	5.90	0.32

The survey used a global 6-point rating scale (Likert Scale) to indicate a range of *very bad *to *very good, strong rejection *to *strong agreement*. * Total number. N refers to the number of trainees quoting. SD refers to standard deviation.

**Table 2:** Essential results of the post-course survey

	N	Range	Mean	SD
I consider a course to train vascular anastomosis techniques as important	10	5.00 - 6.00	5.60	0.70
I consider porcine aorta as suitable to train vascular anastomosis	10	5.00 - 6,00	5.70	0.48
The course was demanding	10	3.00 - 6.00	4.90	0.99
The lecture is important	10	4.00 - 6.00	5.00	0.66
How was time management to lern vascular anastomosis techniques?*	10	1.00 - 2.00	1.80	0.42

The survey used a global 6-point rating scale (Likert Scale) except for (*) to indicate a range of *very bad *to *very good, strong rejection to strong agreement*. (*) Only a 3-point rating scale was used indicating *not enough*, *optimal *and *to long*. N refers to the number of trainees quoting. SD refers to standard deviation.

## Discussion

We have provided here a simple model to impart vascular anastomosis skills to surgical residents using formalin-fixed porcine aortic segments. There are several models used to train vascular anastomosis techniques that have been described in the literature so far [3-17]. However, most of these models address microvascular techniques using an operative microscope, and are thus not suitable to learn large-vessel anastomoses due to the small diameter of the vessels used. However, some of these models may be adopted for a more general usage by changing the size of the particular model. Tubes of different artificial material like silicone, Gore-Tex, latex and PTFE have been used for vascular anastomosis training [3,5,7,11,13]. However, there may be some expense with vascular tube grafts if unsatisfactory amounts are found as leftovers from the operating room. The handling of these artificial materials differs from that of biological materials due to their stiff nature, making tube grafts not the most favourable model for surgical beginners, even though the suturing technique remains the same. Foodstuffs such as vessels chicken wings and legs, turkey necks or breads, animal limbs and organs, or pig hearts, coronaries or omentum vessels are similar to human vascular texture but only suitable for microsurgical purposes [4]. Moreover, some of these models raise ethical concerns since they use human food resources. The presented porcine aorta model comes very close to the vascular texture of humans and does not require any further preparation besides formalin fixation. The aortic segments can be stored in formalin until eventual use.

The model itself was described initially by Greenhalgh and Flack almost 30 years ago and later was extended to an aneurysm anastomotic model [10,18]. The Royal College of Surgeons of England, which is often on the cutting edge of advancements in structured surgical education has devoted time to promoting skills training workshops for their trainees, and has used this model since it was proposed [9]. However, to the best of our knowledge, no evaluation of the model or the skills course segment for training vascular anastomosis techniques using porcine aortas has been published since its initial introduction. Moreover, the initial report by Greenhalgh and Flack and the report of the description of the first anastomosis workshop by the royal College of Surgeons of England remain the only two references of this model in the literature [10,18].

When using the porcine aorta model, it is important to control for the diameter of the segments used. The aortic segments used in this study had a diameter of 2.0-3.0 cm. However, if a smaller-diameter vessel is desired, porcine iliac or subclavian arteries can be used instead. Porcine vessels can be obtained from any urban slaughterhouse without great financial efforts since they are a by-product of slaughtering. Obtaining the material should be only a matter of negotiation. Vascular tissue does not enter the food chain and is therefore not open to ethical concerns. No extra preparation is necessary and the teaching session is therefore easily prepared.

The survey evaluation of the model presented revealed that there is a strong need for vascular anastomosis skills training among young surgical residents within their first two years of training. The model itself was rated overall with high acceptance, and the skills module of only one hour was rated, as being exactly what is needed. However, we acknowledge that a comparison to other models e.g., tube grafts, is warranted in the future to determine rankings among the currently available models. The present manuscript represents to our best knowledge both the first profound description of a teaching course to train surgical residents using porcine aorta and an evaluation of the same by German surgical residents. However, we acknowledge the preliminary character of this evaluation due to its limited group size. We have used the present model to teach basic vascular anastomosis skills for several years. We use individualised verbal feedback during anastomotic suturing and after the anastomosis is performed by discussing every single stitch with the trainee to objectify the grade of anastomosis leakage. However, several other methods to assess the operative performance have been described, such as grading possible anastomotic leakage or the motor handling of the surgical instruments quantitatively [3,5,19]. These methods can be used as required. Laboratory-based surgical skills training, including vascular anastomosis training, has been demonstrated to improve surgical performance substantially [20]. In this context, it has been previously stated that verbal feedback from an expert instructor leads to lasting improvements in technical performance when learning new surgical skills [21]. It is well recognised that surgical performance determines the patient outcomes. Thus, it has been previously stated that operative procedures are 75% decision-making skills and 25% dexterity [3].

Keeping the importance of manual surgical skills in mind, young surgical residents at the University of Marburg, Germany currently pass through a surgical skills curriculum while they are on non-operative units. They perform, among other procedures; ten bench-model based vascular anastomoses prior to performing them on patients in the operating room [16,22]. This exemplary attempt to establish a continuous curriculum for surgical residents is one important step towards improving both quality in surgical education and the satisfaction of the trainees in surgery. The particular model the residents are trained with is not as important as to serve the particular needs of the trainees. The present evaluation showed the need for training in vascular anastomoses according to the young general surgical residents. In recent years, undergraduate medical education in Germany was revised and changed to include more structured curricula, including formal evaluations, due to licensing requirements and the obvious need of reforms. It is now time to transfer these experiences and build nationwide comprehensive structured surgical curricula to accompany surgical residency programs. This need becomes apparent when considering a survey of general surgery residency programs in the United States that revealed that 86% had specific curricula for teaching book knowledge, but only 45% had curricula for single surgical techniques [16,23]. The most important basic surgical skills needed have been already described and have been compulsory in the graduate training program of the Royal Colleges of Surgery of England, among others, for several years [24]. Thus, residency programs can be enhanced and the contentment among the trainees increased with the deployment of suitable and innovative bench models.

## Competing interests

The authors declare that they have no competing interests.

## Authors' contributions

The course for teaching vascular anastomosis skills was introduced several years ago at the University of Munich by CJB who still enthusiastically serves as their primer course instructor. PNK and AK contributed equally to the present manuscript and thus share first authorship. PNK, AK, CJB and KWJ designed the present study including the preparation of the survey. The data collection was done by PNK and AK. MR and WET helped with the analysis and interpretation of the data. PNK and AK wrote the manuscript. The manuscript was critically revised by MT, WET, KWJ and CJB. All authors read and approved the final version of the manuscript.

**Figure 1:**
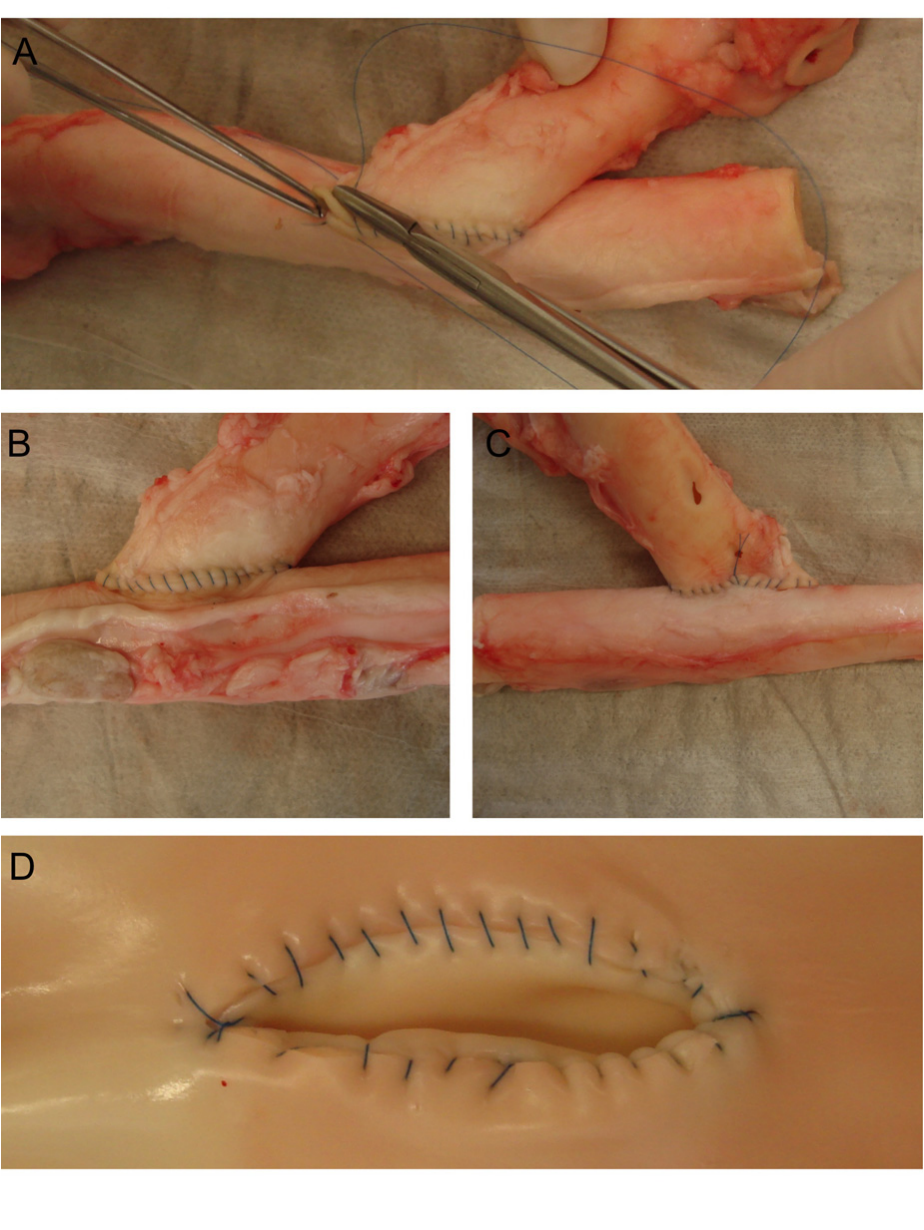
**Representative performance of an end-to-side vascular anastomosis using porcine aorta**. Surgical performance during suturing of the front wall (A). Result of the sutured front and back walls (B and C). The aorta is cut longitudinally for inspection of the endovascular side of the performed anastomosis and to discuss the final result with the surgical trainee (D).
